# Pancreatic Cancer Prognosis, Malnutrition Risk, and Quality of Life: A Cross-Sectional Study

**DOI:** 10.3390/nu14030442

**Published:** 2022-01-19

**Authors:** Kalliopi-Anna Poulia, Dimitra Antoniadou, Panagiotis Sarantis, Michalis V. Karamouzis

**Affiliations:** 1 Laboratory of Dietetics and Quality of Life, Department of Food Science and Human Nutrition, Agricultural University of Athens, 11855 Athens, Greece; lpoulia@gmail.com; 2 Oncology Department of Daily Hospitality, Laiko General Hospital, 11527 Athens, Greece; demiantoniadou@gmail.com; 3 Molecular Oncology Unit, Department of Biological Chemistry, Medical School, National and Kapodistrian University of Athens, 75, M. Asias Street, 11527 Athens, Greece; panayotissarantis@gmail.com

**Keywords:** pancreatic cancer, malnutrition, quality of life, survival, cachexia

## Abstract

The present cross-sectional prospective study (from January 2017 until December 2020) aimed to evaluate the interrelation between nutritional parameters at the initial evaluation with the changes in QOL indicators and the Progression-Free Survival and Overall Survival in 97 patients with pancreatic cancer. Dietary and nutritional risk assessments, and an evaluation of Frailty and Functional Status were performed. Health-related Quality of Life was evaluated with the EORTC QLQ-C30 questionnaire. Nutritional risk, as evaluated by MNA-SF score, was negatively associated with age (*p* = 0.003) and positively associated with dynamometry (*p* = 0.001). In addition, there was a statistically significant difference in the financial difficulties (*p* = 0.049). OS and PFS were found to decrease in patients who reported having increased fatigue (OS *p* = 0.007 and PFS *p* = 0.044). Moreover, higher prevalence of constipation resulted in lower OS and PFS (OS *p* < 0.001 and PFS *p* < 0.001) and increased reporting of gastrointestinal problems during the 3 months was connected with lower survival (OS *p* = 0.017). According to the analysis, our patients were found to have lower OS and PFS, stressing out the necessity to provide early identification and management to symptoms of the disease that could compromise nutritional status. The provision of nutritional advice and guidance is of major importance for patients.

## 1. Introduction

Pancreatic cancer (PC) is characterized by low rates of survival, despite the prolongation of life expectancy and the decrease in mortality in many cancers. According to the recent epidemiological data, pancreatic cancer accounts for as many deaths as cases and it is the seventh leading cause of cancer mortality [1]. Moreover, the incidence and, hence, the mortality rates follow a rather stable course through the years, especially in developed countries, probably reflecting the high prevalence of non-communicable diseases that relate to the pathophysiology of PC, namely diabetes mellitus and obesity, and lifestyle risk factors, i.e., smoking and alcohol intake [2]. Given the fact that breast cancer mortality is decreasing, it was projected that, by 2025, PC will be one of the three leading causes of cancer death in Europe [3].

As mortality rates are significantly high in PC, the effect of the course of the disease on overall wellbeing and the quality of life (QOL) of patients is of great interest. QOL is a concept that incorporates multiple dimensions of self-reported evaluation of wellbeing and functioning. It combines two major components. The ability to perform everyday living activities, reflecting physical, psychological, and social wellness, and the patients’ perception about the level of functionality and the course of the disease [4]. It includes the evaluation of social, emotional, and cognitive functions, sleep quality, endurance in social participation, as well as symptoms of the disease that could have an impact on the self-perceived QOL and financial stability, i.e., constipation and diarrhea [5].

One of the main symptoms of PC, even before diagnosis, is non-volitional weight loss. Cachexia, the multifactorial syndrome that includes muscle and adipose tissue wasting, anorexia and bodyweight reduction is a common feature of PC, resulting in a significant negative impact on the functionality and higher need for supportive care [6]. Cachexia in PC patients is closely connected with higher rates of morbidity and mortality; therefore, the early identification of the problem and the provision of nutritional support is vital for the maintenance of the nutritional status, the muscle mass and the functionality of the patient, the improvement of the nutritional intake, the reduction in the prevalence of toxicity and discontinuation of the treatment, factors that negatively affect the overall efficacy of the therapeutic management and deteriorate the patients’ QOL [7,8].

The present study aimed to evaluate the interrelation between nutritional parameters at the initial evaluation with the changes in QOL indicators and the Progression-Free Survival (PFS) and Overall Survival (OS) in a group of patients with pancreatic cancer.

## 2. Materials and Methods

### 2.1. Study Design and Subjects

A cross-sectional prospective study was conducted from January 2017 until December 2020 in the Oncology Day Clinic of Laikon General Hospital of Athens. Ninety-seven patients (49 men and 48 women, mean age 68.15 ± 9.44 years) with the diagnosis of pancreatic cancer were consecutively enrolled during their introductory visit for chemotherapy (Table 1), after being informed of the aim of the study and the provision of written consent. The study protocol was approved by the Medical Ethics Research Committee of the Laikon General Hospital of Athens.

### 2.2. Anthropometric Measurements

Anthropometric measurements were performed with the subjects wearing light clothing without shoes. Bodyweight and height were measured by the same researcher, at the time of recruitment and the follow-ups every three months according to the study protocol, using a scale and a wall-mounted stadiometer, to the nearest 0.5 kg and 0.5 cm. If anthropometric measurements were not feasible (e.g., in case of a patient unable to stand due to fatigue or pain), calf circumference and knee height were measured with a non-stretched tape. Body Mass Index (BMI) was computed as weight (in kilograms) divided by height (in meters squared). The percentage of unintentional weight loss over the last 6 months was calculated following patient reports.

Mid-arm circumference (MAC) and Triceps Skinfold Thickness (TSF) were measured for all participants during the first visit and in the follow-ups. MAC was assessed using a non-stretchable tape measure to the nearest 0.1 cm at the midpoint between the olecranon process and the acromion when the subject was standing with their arm hanging relaxed next to the body and their palm facing the thigh. TSF was then measured with a caliper (Lange, Cambridge, MD, USA) at the same point. To assess muscle mass, including and excluding bone mass, MAMC was estimated using a standard formula: MAMC = MAC − (3.1415 × TSF) [9].

Handgrip strength was measured on the dominant hand with a Jamar Analogue Hand Dynamometer with the participants seated, their elbow by the side, and a neutral wrist position. The higher measurement out of the three attempts was recorded [10]. The cut-offs of the European Working Group on Sarcopenia in Older People 2 (EWGSOP2) were used for men and women, namely 27 kg for men and 16 kg for women. Dynamopenia was defined according to the EWGSOP2 criteria [11].

### 2.3. Nutritional Risk

Nutritional risk screening was performed with the Mini Nutritional Assessment Screening Form (MNA-SF). The MNA-SF contains six questions evaluating weight loss, appetite, mobility, psychological stress, neuropsychological problems and BMI. It was initially designed for elderly patients, but it has been validated in other populations as well. MNA-SF scores range from 0 to 14 and patients are categorized as having “normal nutritional status (scores from 12 to 14), at nutritional risk (scores from 8 to 11) or malnourished (scores from 0 to 7) [12,13].

During the study, all the patients were assessed by a clinical dietitian and received advice on nutrition-related issues, according to each patient’s needs. Moreover, all the patients received nutritional advice regarding the possible side effects of the treatment and the management of the nutrition-related ones. Patients at risk of malnutrition or malnourished were also provided with appropriate nutritional supports, to ensure sufficient nutrient and energy intake.

### 2.4. Frailty and Functional Status

The presence of frailty in the initial assessment and the entry in the study was assessed with the Clinical Frailty scale (CFS) [14]. The CFS ranges from 1 (very fit) to 9 (terminally ill), as described elsewhere. The Charlson Comorbidity Index (CCI) was used to assess comorbidities. The CCI includes 17 questions regarding the presence of other clinical conditions that could have an impact on the overall health status and the risk of mortality [15]. Finally, performance status and the need for assistance were evaluated with the Katz Index, with the score ranging from 0 to 6, with high scores indicating a high level of independence and low scores indicating a need for help in activities such as dressing up, bathing, feeding, toileting, transferring and continence [16].

### 2.5. Health-Related Quality of Life (HQoL)

Health-related Quality of Life (HQoL) was evaluated with the EORTC QLQ-C30 questionnaire developed by the European Organization for Research and Treatment of Cancer (EORTC), which is composed of multi-item functional subscales: physical, emotional, role, social and cognitive functioning; three multi-item symptom scales measuring pain, fatigue and emesis; a global health/quality of life subscale; and six single items assessing financial impacts and symptoms such as dyspnea, sleep disturbance, appetite, diarrhea and constipation [17]. Patients’ responses were evaluated and summarized according to standard HRQoL nomenclature [18]. Measurements of HQoL were repeated every three months to all patients who continued chemotherapy.

### 2.6. Statistical Analysis

Continuous variables were presented with descriptive statistics and various measures, while categorical variables were presented with counts and percentages. Possible associations between categorical variables were tested using Fisher’s exact test, while associations between categorical and continuous variables using the Mann–Whitney or Kruskal–Wallis test as appropriate. All continuous variables were tested for correlations using Spearman’s correlation coefficient. Correlations between variables with repeated measurements were calculated using the repeated measures correlation technique in R.

For the variables with repeated measurements, i.e., HQoL, the differences as a percent of change from baseline were computed for the 3-month measurements. The percent of change from baseline was then categorized into 3 categories (<−30%, −30–30% and >30%), and in some cases, into 2 categories (<−30% and ≥−30%).

Impact on survival was tested using 3 different time-to-event endpoints: Overall Survival (OS), Progression-Free Survival (PFS) and Progression-Free Survival—FU (PFSFU). OS was calculated as the time from diagnosis to death (event) or the last date of contact that the participant was known to be alive (censored). PFS was calculated as the time from diagnosis to documented progression (event), death (event) or the last date of contact that the participant was known to be alive (censored). PFS_FU was calculated as the time from diagnosis to documented progression (event), death (event) or the last date of contact that the participant was known to not have had progression (censored).

Kaplan–Meier curves were plotted for the categorical variables, while the product-limit method was used to calculate the median survival (and 95% CI). Log-rank test *p*-values were also calculated to compare the survival distributions of the different categories. Univariate Cox regressions were run for both continuous and categorical variables. Those found to be statistically significant for the set representing the categorized change from baseline QoL scores were also checked in a multivariate setting controlling for basic characteristics (age, gender, weight loss and BMI).

IBM SPSS Statistics Version 26 and R version 4.0.5 (3 March 2021) were used for performing all the statistical analyses described above.

## 3. Results

A total of 97 patients (49 men) with pancreatic cancer (*n* = 76) or pancreatic cancer with hepatobiliary metastasis (*n* = 21) were included in the study, with a mean age of 68.1 ± 9.4 years. Most patients reported that they were independent, as evidenced by the Katz score of 88.5%, with 8% reporting to be totally dependent. According to the CFS, 81.3% of the patients were managing well or were fit, and 18.3% were mildly frail or vulnerable. None of the patients were frail. Seventy-six patients were diagnosed with pancreatic cancer (C25), 21 had pancreatic and hepatobiliary carcinomas (C22-24-25) and 17% of the patients had undergone surgery prior to chemotherapy. The mean BMI was 24.2 ± 3.5 m/kg^2^. The mean weight loss percentage at three and six months prior to the initiation of therapy was 8.0 ± 9.5% and 12.45 ± 8.99% accordingly. Overall, 64.5% of the patients reported that they had never received nutritional advice prior to the day of inclusion in the study and 32 had at least a session with a nutritionist or dietitian. Interestingly, 80.3% of the patients were either at high risk or malnourished according to the MNA-SF score at the time of recruitment (Table 2). Moreover, 75% of the male and 76.5% of the female patients were found to be dynamopenic, based on the criteria of EWGSOP2 [11]. Nutritional risk, as evaluated by the MNA-SF score, was negatively associated with age (r = −0.320, *p* = 0.003) and CCI (r = −0.307, *p* = 0.004) and positively associated with dynamometry (r = 0.387, *p* = 0.001).

The evaluation of the HQoL questionnaires revealed that the perception of the HQoL among patients was compromised and remained as such during their treatment, with a tendency of improvement that did not reach significance levels for the patients reaching 9 months of treatment. Although the differences in most of the measured aspects of QoL did not change in the examined period, there was a statistically significant difference in the financial difficulties that the patients reported over the duration of the study, which almost doubled in the nine months of follow-up for the survivors (0.23 ± 0.30 at baseline vs. 0.45 ± 0.35 at nine months, *p* = 0.049) (Table 3). As for possible correlations of the HQoL with nutritional parameters, the dynamopenic patients had significantly lower hQoL compared to the non-dynamopenic patients (71.35 ± 27.55 vs. 56.12 ± 24.58, *p* = 0.011) at baseline.

**Figure 1:**
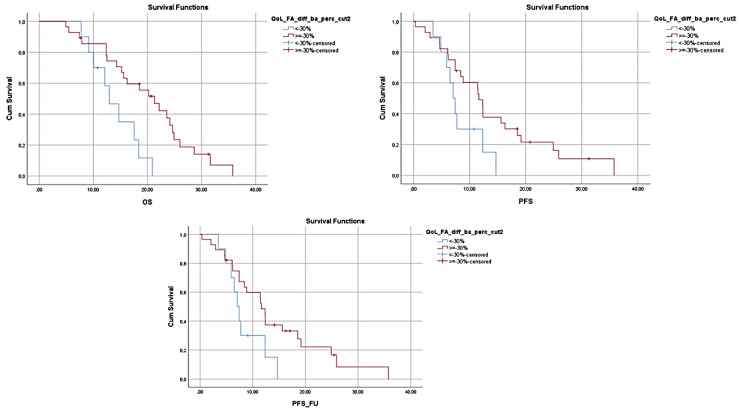
OS, PFS and PFS_FU for patients reporting >30% increases in fatigue at 3 months vs. the patients reporting ≤30% increases in fatigue. OS and PFS were found to decrease in patients who reported having increased fatigue (OS *p* = 0.007 and PFS *p* = 0.044).

**Figure 2:**
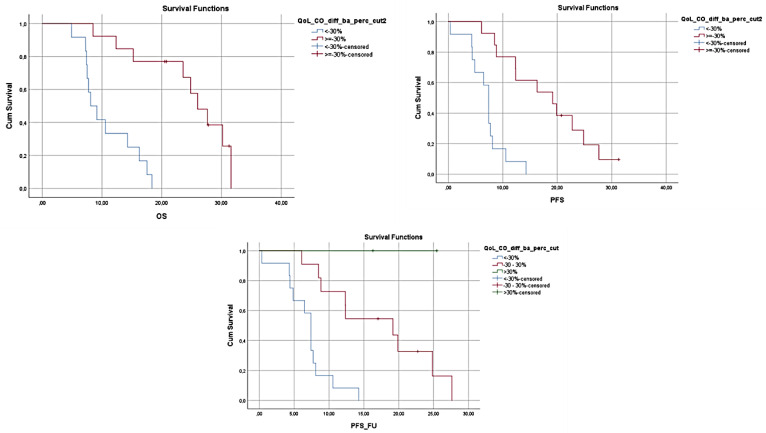
OS, PFS and PFS_FU for patients reporting >30% increases in constipation at 3 months vs. the patients reporting ≤30% increases in the same symptom. Higher prevalence of constipation resulted in lower OS and PFS (OS *p* < 0.001 and PFS *p* < 0.001).

**Figure 3:**
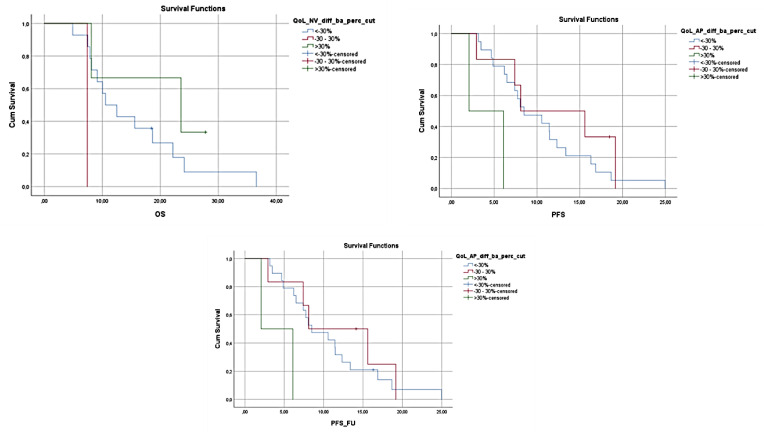
OS for patients reporting a >30% increase in nausea and vomiting, PFS and PFS-FU for patients reporting a >30% increase in anorexia at 3 months vs. the patients reporting ≤30% increase in nausea and vomiting or anorexia, respectively. Increased reporting of gastrointestinal problems during the 3 months related to lower survival (OS *p* = 0.017).

At the same time OS and PFS were found to decrease in patients who reported having increased fatigue (OS 12.97, 95% CI 9.346–16.587 vs. 21.3, 95% CI 16.024–26.576, *p* = 0.007 and PFS 7.067, 95% CI 5.672–8.461 vs. 11.633, 95% CI 10.703–12.564, *p* = 0.044), and the presence of increased fatigue was connected with an increased risk of mortality compared to the patients who did not report similar limitations (HR 3.159, 95% CI 1.317–7.579, *p* = 0.010). Moreover, increased reporting of gastrointestinal problems during the 3 months was also connected with lower survival. More specifically, an increase in nausea and vomiting was found to be related to lower OS (24.833, 95% CI 0.000–48.318 vs. 10.567, 95% CI 6.044–15.089, *p* = 0.017), and higher prevalence of constipation resulted in lower OS and PFS (OS 26.000, 95% CI 21.703–30.297 vs. 8.100, 95% CI 5.724–10.476, *p* < 0.001 and PFS 7.400, 95% CI 6.440–8.360 vs. 19.167, 95% CI 10.359–27.974, *p* < 0.001), with patients reporting side effects that could limit nutrient intake and compromise nutritional status having significantly higher risk of mortality (Nausea and Vomiting HR 31.156, 95% CI 1.409–68.901, *p* = 0.029, Constipation HR 10.347, 95% CI 2.801–38.233, *p* < 0.001) or progression of the disease (Constipation HR 7.520, 95% CI 2.484–22.769, *p* < 0.001). In Figure 1, Figure 2 and Figure 3, the Kaplan–Meier plots of the OS, PFS, and PFS_FU, according to the differences of the above-mentioned symptoms at 3 months compared to baseline, are presented.

## 4. Discussion

According to the analysis of our patients, almost 85% of them reported having a high risk of malnutrition or established malnutrition, significant weight loss, reaching 12% of their usual body weight at 6 months, and functional disabilities, with low handgrip strength and self-reported fatigue. The nutritional abnormalities and cancer cachexia are common problems in patients with pancreatic cancer, as the metabolic and hormonal alterations, the anorexic effects of the tumor and the problematic nutrient intake and absorption, the accelerated metabolism and inflammation result in a negative energy balance, affecting mostly the muscle mass and functionality [19]. Mean weight loss was found to be very significant, exceeding 8% at 3 months, and 12% at 6 months prior to the initial evaluation, a common characteristic of patients with PC, and often the leading alerting cause before diagnosis. It is of value to mention that despite the nutritional abnormalities, most of them did not receive nutritional advice prior to the initiation of the chemotherapy [20,21]. Moreover, the provision of nutritional advice and guidance from all of the medical team is of major importance for the patients, in case a dietitian is not available. The role of the nursing staff should be enhanced, especially at the end of the treatment, where the patients are more receptive to hearing about nutrition and health [22,23].

Improving and maintaining muscle mass and functionality can have a major impact in terms of quality of life, independence, and better management of the disease. Patients with lower handgrip strength and dynamopenia reported lower HQoL. Studies on other types of cancer, i.e., colorectal cancer, identified the maintenance of skeletal muscle mass and functionality as an important factor affecting QoL [24]. On the other hand, added to the low muscle mass, symptoms related to lower nutritional intake, i.e., nausea and vomiting and constipation, were found to affect the prognosis of the patients, as they result in either limited nutrient intake or a higher possibility of discontinuation of the treatments due to higher toxicity [25]. According to the analysis, our patients were found to have lower OS and PFS, highlighting the necessity to provide early identification and management of symptoms of the disease that could compromise nutritional status.

One other parameter of the HQoL questionnaire that was also found to be relevant to cancer patients is the burden of financial difficulties that follow the patients throughout the treatments. Surviving patients reported significantly higher difficulty in managing financial issues. The so-called “financial toxicity” is a common problem in patients with cancer, especially in the older ones, resulting in significant negative effects in terms of their hQoL and overall survival [26,27]. Financial toxicity can be triggered by baseline factors, i.e., the socioeconomic status of the patients, the overall insurance coverage and the provision of palliative and supportive care during the disease. Moreover, the type of cancer, the symptoms and the adverse effects of the treatments alongside the need for care during the treatments can be negatively associated with the economic burden of the disease. [28] During the last decade, Greece faced a severe economic crisis, with high unemployment, increased “out of the pocket money” for health and strict austerity measures that had a negative impact on the quality of public health system services [29]. Therefore, financial toxicity could be easily developed, especially in cancer patients, who usually present limited ability to work and function during and even after the end of the treatments, compared to the healthy population. [28,30].

According to the available data, nutrition counseling and advice can improve the body weight and energy intake of patients with cancer; however, there is a need for well-designed Randomized Controlled Trials (RCTs) focusing on the effects of dietary interventions on nutrition-focused clinical outcomes, such as reaching nutritional needs and assuring the intake of specific macro and micronutrients, to ensure the best possible outcomes for patients.

## Figures and Tables

**Table 1:** Τhe chemotherapeutic regimens for the treatment of patients.

Number of Patients	Chemotherapy Regimen
67	Nab-Paclitaxel plus Gemcitabine
10	Carboplatin plus Gemcitabine
16	Gemcitabine
3	FOLFIRINOX
1	FOLFOX

**Table 2:** Baseline characteristics of the sample.

Characteristics	*n* = 97
Sex	
*Men*	49
*Women*	48
Age (years)	68.1 ± 9.4
Height (m)	1.67 ± 0.09
Body weight (kg)	68.2 ± 13.3
BMI (kg/m^2^)	24.2 ± 3.5
ICD-10, *n* (%)	
*C25*	76 (77.8%)
*C22-24-25*	21 (21.6%)
Clinical Frailty scale	
*1 (very fit)*	10.50%
*2 (well)*	23.30%
*3 (Managing well)*	47.70%
*4 (Vulnerable)*	16.30%
*5 (Mildly Frail)*	2.30%
*6–9 (Moderately frail—terminally ill)*	0%
Katz Score	
*0 (dependent)*	8%
*1*	2.3%
*2*	1.1%
*6 (independent)*	88.5%
Surgery prior to chemotherapy	
*Yes*	17%
*No*	83%
Reported involutional weight loss (kg)	
*3 months*	6.5 ± 7.3
*6 months*	10.2 ± 7.4
% of weight loss	
*3 months*	8.0 ± 9.5
*6 months*	12.45 ± 8.99
MNA-SF score *n* (%)	
*Good nutritional status*	8 (8.9%)
*Risk of malnutrition*	35 (36.8%)
*Malnourished*	43 (44.3%)
*Missing data*	11 (11.3%)
Nutrition advice prior to chemotherapy	
*Yes*	32 (33%)
*No*	63 (64.9%)
*Missing*	2
Dynamopenia *n* (%)	
*Men*	27 (75%)
*Women*	26 (76.5%)
*Missing values*	27

**Table 3:** HQoL questionnaires results.

HQoL Issue	HQoL-Baseline (%) (Mean ± SD) *n* = 91	HQoL-3 Months (Mean ± SD) *n* = 71	HQoL-6 Months (Mean ± SD) *n* = 38	HQoL-9 Months (Mean ± SD) *n* = 17	*p*
Global HQoL	0.59 ± 0.25	0.62 ± 0.25	0.59 ± 0.25	0.73 ± 0.20	NS
Functional Issues					
*Physical functioning*	0.71 ± 0.24	0.69 ± 0.25	0.71 ± 0.24	58.67 ± 0.32	NS
*Role functioning*	0.62 ± 0.35	0.64 ± 0.36	0.58 ± 0.37	0.66 ± 0.37	NS
*Emotional functioning*	0.71 ± 0.27	0.71 ± 0.28	0.60 ± 0.33	0.64 ± 0.21	NS
*Cognitive functioning*	0.84 ± 0.24	0.82 ± 0.23	0.77 ± 0.25	0.77 ± 0.31	NS
*Social Functioning*	0.82 ± 0.28	0.75 ± 0.29	0.65 ± 0.35	0.56 ± 0.41	NS
Symptom scales					
*Fatigue*	0.42 ± 0.31	0.40 ± 0.30	0.44 ± 0.29	0.42 ± 0.34	NS
*Nausea and vomiting*	0.12 ± 0.24	0.10 ± 0.23	0.10 ± 0.21	0.07 ± 0.13	NS
*Pain*	0.28 ± 0.28	0.27 ± 0.28	0.37 ± 0.28	0.30 ± 0.33	NS
*Dyspnea*	0.22 ± 0.3	0.25 ± 0.28	0.29 ± 0.31	0.23 ± 0.20	NS
*Insomnia*	0.37 ± 0.36	0.31 ± 0.34	0.41 ± 0.36	0.33 ± 0.33	NS
*Appetite*	0.29 ± 036	0.19 ± 0.30	0.28 ± 0.38	0.31 ± 0.42	NS
*Constipation*	0.24 ± 0.31	0.20 ± 0.29	0.31 ± 0.36	0.12 ± 0.26	NS
*Diarrhea*	0.14 ± 0.25	0.18 ± 0.29	0.17 ± 0.23	0.22 ± 0.33	NS
Financial	0.23 ± 0.30	0.32 ± 0.31 *	0.42 ± 0.34 *	0.45 ± 0.35 *	0.049

* Statistical significance, *p* < 0.05.

## Data Availability

The data presented in this study are available on request from the corresponding authors.
